# Deep-level defects in n-type GaAsBi alloys grown by molecular beam epitaxy at low temperature and their influence on optical properties

**DOI:** 10.1038/s41598-017-13191-9

**Published:** 2017-10-09

**Authors:** Łukasz Gelczuk, Jan Kopaczek, Thomas B. O. Rockett, Robert D. Richards, Robert Kudrawiec

**Affiliations:** 1 Faculty of Microsystem Electronics and Photonics, Wrocław University of Science and Technology, Janiszewskiego 11/17, 50-372 Wroclaw, Poland; 2 Faculty of Fundamental Problems of Technology, Wrocław University of Science and Technology, Wybrzeże Wyspiańskiego 27, 50-370 Wrocław, Poland; 3 Department of Electronic and Electrical Engineering, University of Sheffield, Sheffield, S1 3JD United Kingdom

## Abstract

Deep-level defects in n-type GaAs_1−*x*_Bi_*x*_ having 0 ≤ *x* ≤ 0.023 grown on GaAs by molecular beam epitaxy at substrate temperature of 378 °C have been injvestigated by deep level transient spectroscopy. The optical properties of the layers have been studied by contactless electroreflectance and photoluminescence. We find that incorporating Bi suppresses the formation of GaAs-like electron traps, thus reducing the total trap concentration in dilute GaAsBi layers by over two orders of magnitude compared to GaAs grown under the same conditions. In order to distinguish between Bi- and host-related traps and to identify their possible origin, we used the GaAsBi band gap diagram to correlate their activation energies in samples with different Bi contents. This approach was recently successfully applied for the identification of electron traps in n-type GaAs_1−*x*_N_*x*_ and assumes that the activation energy of electron traps decreases with the Bi (or N)-related downward shift of the conduction band. On the basis of this diagram and under the support of recent theoretical calculations, at least two Bi-related traps were revealed and associated with Bi pair defects, i.e. (V_Ga_+Bi_Ga_)^−/2−^ and (As_Ga_+Bi_Ga_)^0/1−^. In the present work it is shown that these defects also influence the photoluminescence properties of GaAsBi alloys.

## Introduction

GaAs_1−*x*_Bi_*x*_, containing a few percent Bi (dilute bismides), grown on GaAs have attracted a lot of attention in recent years due to their unusual properties including a strong band gap reduction (~84 meV/%Bi) and a large spin-orbit splitting^1,2^, which can supress Auger processes if the splitting is larger than the band gap (i.e., Δ_SO_ > E_g_)^3^, as well as a type I band gap alignment at the GaAsBi/GaAs interface^4,5^. Because of this, such alloys are promising candidates for optoelectronic devices^6,7^. This material system is also complementary to the well-studied dilute nitride alloy GaAs_1−*x*_N_*x*_. Incorporating Bi into GaAs perturbs the valence band, whereas N in GaAs perturbs the conduction band^8,9^. Therefore, due to strong carrier scattering at localized states, N alloying mainly affects the electron density and mobility, while Bi alloying mainly affects the hole density and mobility in GaAs^10^.

The crystal quality of III-V alloys is highly affected by the growth temperature. A low-temperature (LT) growth is undesirable as it typically leads to increased defect densities and optical quality degradation. However, the efficient incorporation of Bi requires low temperatures — typically below 400 °C — and nearly stoichiometric growth conditions — i.e. V/III flux ratio ~1 — due to a large immiscibility between Bi and GaAs^11,12^. The Bi flux has to be accurately controlled, as excess Bi during growth may accumulate and form Bi droplets, while insufficient Bi flux may cause surface roughening due to a reduced surfactant effect^11–13^. It is generally known that growing GaAs at temperatures significantly lower than the optimal growth temperature (580–600 °C) favours the increase of point defect concentrations, such as As-antisities (As_Ga_), As-interstitials (As_i_) and Ga vacancies (V_Ga_)^12,14^. Indeed, GaAs is found to have defect concentrations that increase strongly as the growth temperature is decreased^15^. Temperature dependent Hall effect measurements of undoped GaAs layers grown by molecular beam epitaxy (MBE) at 300 °C and 400 °C, show that the layers are n-type with high donor concentrations of 3 × 10^18^ cm^−3^ and 2 × 10^17^ cm^−3^, respectively^16^. The dominant deep donor level in LT-GaAs has been shown to involve As_Ga_ with an activation energy of 0.65 eV below the conduction band^14,16^. However, incorporating Bi into GaAs at low temperatures enhances surface migration, thus reducing the density of Ga and/or As-related defects, but introducing Bi-related defects at the same time. Due to a strong tendency for Bi to surface segregate and the fact that it is isoelectronic with As, growth of GaAs_1−*x*_Bi_*x*_ alloys typically involves a Bi surfactant layer, which acts to smooth the growth surface and improves the electro-optical properties of the material^17,18^. Photoluminescence (PL) measurements of GaAs_1−*x*_Bi_*x*_ films indicate that dilute amounts of Bi (*x* < 0.025) improve the crystal quality, but larger Bi concentrations cause degradation of the material quality, probably due to Bi-related defects^12^. Recent deep level transient spectroscopy (DLTS) studies of n-type GaAs_1−*x*_Bi_*x*_ layers with 0 ≤ *x* ≤ 0.012 revealed a significant reduction of the total trap concentration by more than a factor of 20, in comparison with GaAs layers grown at the same low temperature (330 °C)^19,20^. The dominant deep level defect in these layers was suggested to be an As_Ga_-related complex defect, with an activation energy of about 0.65 eV, as expected for MBE growth at this temperature. However, Bi incorporation can also introduce Bi-related defects such as the Bi-antisite (Bi_Ga_). The Bi_Ga_ double donor defect was previously observed by electron-spin resonance (ESR) in lightly Bi-doped GaAs grown by the liquid encapsulated Czochralski method to have the single ionized 0/+ level at 0.35–0.5 eV below the conduction band^21^.

In this paper, deep-level defects are investigated by DLTS in n-type GaAs_1−*x*_Bi_*x*_ layers having 0 ≤ *x* ≤ 0.023 grown on GaAs substrates by molecular beam epitaxy (MBE). In the experiment, four Bi-containing samples and one GaAs reference sample grown at the same low temperature (378 °C) are investigated. Additionally, contactless electroreflectance (CER) and photoluminescence (PL) techniques are used to study the Bi-dependence of the energy gap and defect-related emission characteristics of the GaAs_1−*x*_Bi_*x*_ layers, respectively. The possible origin of deep electron traps is analyzed in detail with the use of the band gap diagram concept^22^, which has recently been successfully applied for the identification of traps in as-grown and annealed GaAs_1−*x*_N_*x*_ layers grown by MBE^23^ and MOVPE^24^. It enabled us to distinguish between GaAs-like native defects or impurities and Bi-related defects in GaAs_1−*x*_Bi_*x*_ layers. To our knowledge, no direct confirmation of the existence of Bi-related defects executed by DLTS experiments and consistent with theoretical calculations of the predicted positions of defect energy levels in GaAsBi alloys have yet been done.

## Results and Discussion

### Structural and electrical properties

**Figure 1:**
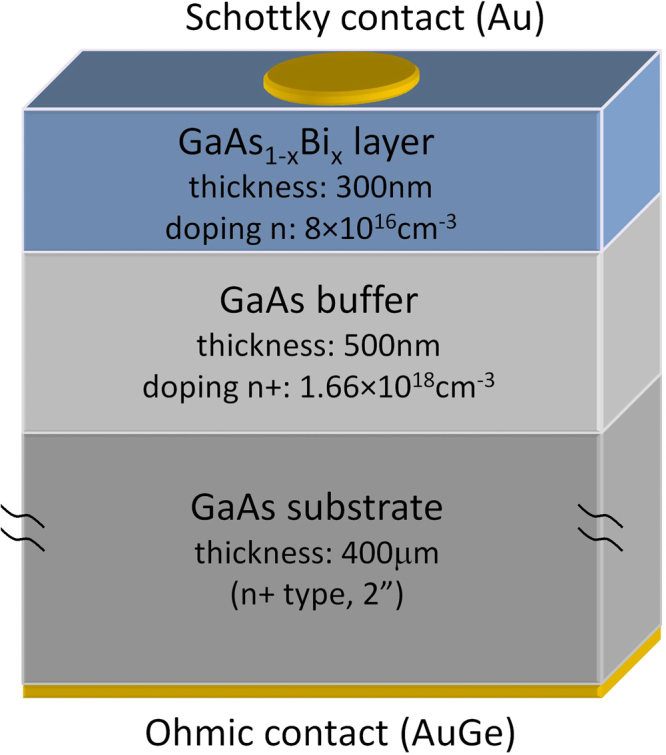
Schematic diagram of the GaAs_1−*x*_Bi_*x*_/GaAs heterostructures with metallic contacts used for DLTS measurements.

Figure 1 shows schematic diagram of the studied GaAs_1−*x*_Bi_*x*_/GaAs layer structure. Four n-type GaAs_1−*x*_Bi_*x*_ epitaxial layers with approximately 0.8%, 1.2%, 1.7% and 2.3% Bi were investigated. Additionally, a 500 nm thick n-GaAs epitaxial layer, grown on a GaAs substrate at the same low temperature (378 °C), was used as a reference sample. The GaAs_1−*x*_Bi_*x*_ epilayer compositions were determined from X-ray diffraction (XRD) rocking curves of the symmetrical (004) reflection, using the Cu Kα_1_ line. All the samples present good crystalline quality with slightly broadened GaAs_1−*x*_Bi_*x*_ layer peaks as compared to their GaAs peaks and clearly visible, strong Pendellösung fringes. The band gap energy of the epilayers was determined from CER spectra by fitting the experimental data with the well-known Aspnes’s formula^25^. The observed band gap reduction with increasing Bi content (see Table 1) is in a good agreement with reported experimental and theoretical data^4^, as well as the band anticrossing (BAC) model^8^.

**Table 1:** Sample properties, i.e. bismuth content (*x*), band gap energy (*E*_*g*_), net doping concentration (*N*_*D*_), and total electron trap concentration (*N*_*T*_^*total*^) of the GaAs_1−*x*_Bi_*x*_ layers, as obtained from room temperature XRD, CER, CV and temperature dependent DLTS measurements, respectively.

Sample	Bi content *x* (%)_XRD_	Band gap energy *E*_*g*_ (eV)_CER_	Net doping concentration *N*_*D*_ (cm^−3^)_C-V_	Total trap concentration *N*_*T*_^*total*^ (cm^−3^)_DLTS_
uSTF7J	0.0	1.42	2.4 × 10^17^	1.8 × 10^16^
uSTF7G	0.8	1.36	1.1 × 10^17^	3.1 × 10^15^
uSTF7E	1.2	1.34	9.3 × 10^16^	5.2 × 10^15^
uSTF7D	1.7	1.29	7.0 × 10^16^	1.2 × 10^15^
uSTF7B	2.3	1.24	4.8 × 10^16^	1.5 × 10^14^

In order to determine the doping concentration in the GaAs_1−*x*_Bi_*x*_ epilayers, a few separate samples comprising a 1000 nm Si-doped GaAs layer were grown on a semi-insulating GaAs substrates at a GaAs growth rate of about 0.625 ML/s, using different silicon cell temperatures. On the basis of Hall effect measurements, it was estimated that a doping concentration of about 8 × 10^16^ cm^−3^ should be achieved in all of the studied samples for a silicon cell temperature of about 1090 °C. However, capacitance versus voltage (C-V) measurements at 300 K revealed that the net doping concentration in the GaAs reference sample is much higher than the predicted value and equal to about 2.4 × 10^17^ cm^−3^. Moreover, as the Bi content increases, the doping concentration is distinctly reduced and it finally attains almost an order of magnitude lower value for 2.3% Bi, as was shown in Table 1. This well-known phenomenon is related to the formation of acceptor-like defects introduced through the incorporation of Bi atoms. The existence of Bi-induced acceptor levels, whose density increases with Bi concentration, leads to a strong compensation of the free electron density, thus giving rise to p-type conductivity of nominally undoped GaAs_1−*x*_Bi_*x*_^26,27^ or reducing n-type conductivity of InP_1−*x*_Bi_*x*_^28,29^ alloys. Bi complexes or clusters involving two or more Bi atoms have been experimentally evidenced as the most likely candidates to form such acceptor levels^29,30^.

### DLTS and LDLTS results

**Figure 2:**
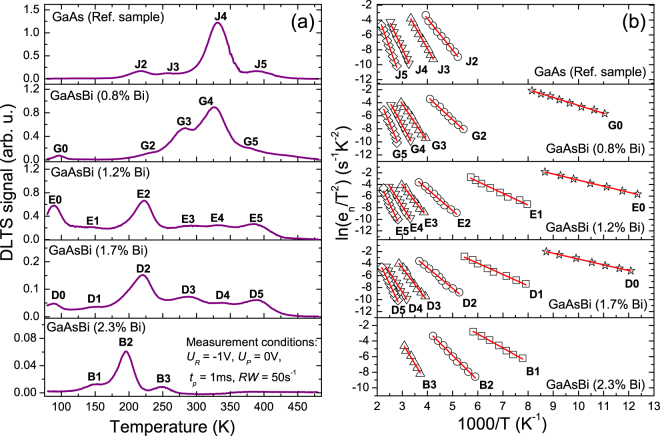
DLTS spectra obtained for n-type GaAsBi layers of various Bi contents grown on GaAs substrates: (**a**) standard DLTS spectra taken with *U*_*R*_ = −1 V, *U*_*P*_ = 0 V, *t*_*P*_ = 1 ms, rate window RW = 50 s^−1^, and (**b**) Arrhenius plots of the revealed electron traps (solid lines represent best fits to the experimental data).

Standard DLTS spectra for the GaAs reference layer and four GaAs_1−*x*_Bi_*x*_ layers of different bismuth concentrations are shown in Fig. 2a. The spectra were recorded within the 80–480 K temperature range. In order to ensure that all the traps are filled up during the DLTS measurements, a steady state small reverse bias voltage (*U*_*R*_) of −1 V and a filling pulse voltage (*U*_*P*_) of 0 V with the width of the filling pulses (*t*_*p*_) set to 1 ms, were chosen as the bias conditions. The emission rate window (RW) was equal to 50 s^−1^.

As illustrated in Fig. 2a, DLTS spectra reveal the presence of four positive peaks, labeled J2, J3, J4 and J5, which correspond to electron emitting levels in the upper half of the band gap in the GaAs reference sample. On the other hand, five (G0 and G2 to G5), six (E0 to E5), six (D0 to D5) and three (B1 to B3) electron traps were detected in the GaAs_1−*x*_Bi_*x*_ layers with 0.8%, 1.2%, 1.7% and 2.3% Bi content, respectively. It is clearly visible that the incorporation of Bi suppresses the formation of GaAs-like defects, thus reducing the total trap concentration (see Table 1), which confirms previous results^12,17,19,20^. However, new electron traps appear in the GaAs_1−*x*_Bi_*x*_ layers, which are not observed in the reference sample, and therefore they are obviously attributed to Bi incorporation into the GaAs host.

**Figure 3:**
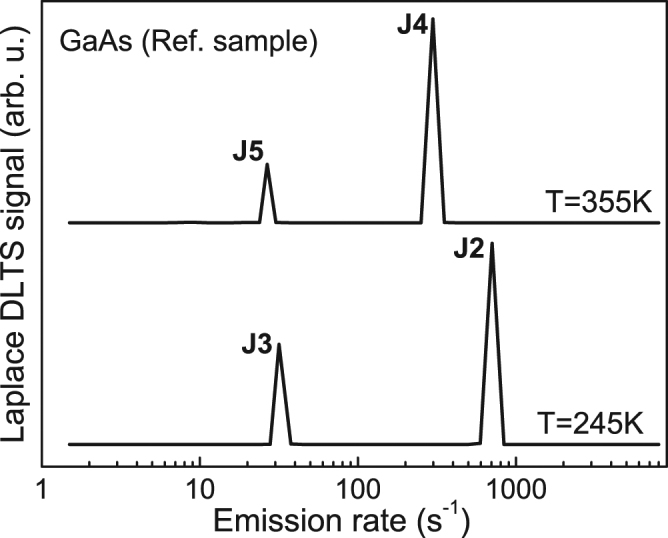
Exemplary LDLTS spectra recorded for the majority peaks J2, J3 and J4, J5 of Fig. 2a in the GaAs reference sample at 245 K and 355 K, respectively.

For a better resolution of deep electron traps in the GaAs_1−*x*_Bi_*x*_ layers, Laplace DLTS (LDLTS) measurements were also performed. LDLTS is a powerful tool for thermal emission studies, because of its significantly improved spectral resolution compared to the standard DLTS technique^31^. Under ideal conditions, LDLTS can resolve defect levels with emission rates differing by a factor of 2. Figure 3 shows exemplary high-resolution LDLTS spectra, recorded for the GaAs reference sample at two different temperatures. As shown in Fig. 3 each of the majority DLTS peaks of Fig. 2a (i.e. traps J2, J3, J4 and J5) corresponds to one dominant, sharp LDLTS peak, which is in agreement with the mono-exponential emission process expected for a well-defined single energy level related to a point defect.

The thermal activation energy (*E*_*a*_ = *E*_*C*_ − *E*_*T*_) — i.e. the deep energy level position (*E*_*T*_) in the band gap in relation to the bottom of the conduction band (*E*_*C*_) — and the apparent capture cross-section (*σ*_*n*_) of electron traps, were determined on the basis of the detailed balance equation^32^:

1 $${e}_{n}={\sigma }_{n}{v}_{th}{N}_{C}\,\exp [-\,{E}_{a}/{k}_{B}T],$$

where *e*_*n*_ is the thermal emission rate for electrons from a deep energy level into the conduction band at temperature *T*, *v*_*th*_ is the thermal velocity of the electrons, *N*_*C*_ is an effective density of states in the conduction band and *k*_*B*_ is Boltzmann’s constant. Considering that *v*_*th*_ ∝ *T* ^1/2^ and *N*_*C*_ ∝ *T* ^3/2^ and assuming temperature independence for the capture cross section, equation (1) becomes a linear equation in the ln(*e*_*n,p*_/*T* ^2^) vs. 1000/*T* plot, called the Arrhenius plot. The thermal activation energy and capture cross section for each deep-level defect are determined from the slope and intercept values of the corresponding Arrhenius plot. In the standard approach, Arrhenius plots are obtained by measuring a shift in the DLTS temperature peak position as a function of an emission rate, whereas in high-resolution Laplace DLTS analysis, the emission rate peak position is measured at various fixed temperatures.

The concentration (*N*_*T*_) of each deep-level defect was determined from the magnitude of the DLTS peak (*ΔC*), using the formula^32^:

2 $${N}_{T}=2{N}_{D}\frac{{\rm{\Delta }}C}{C({U}_{R})}\{\frac{{W}^{2}({U}_{R})}{{(W({U}_{R})-\lambda )}^{2}-{(W({U}_{P})-\lambda )}^{2}}\},$$

where *N*_*D*_ is the net doping concentration, $C({U}_{R})$ is the capacitance at quiescent reverse bias, $C({U}_{P})$ is the capacitance at the filling pulse voltage and *W* is the depletion width, which are determined from C-V measurements at the temperature of the DLTS peak, while

3 $$\lambda ={\{\frac{2{\varepsilon }_{r}{\varepsilon }_{0}}{{e}^{2}{N}_{D}}({E}_{F}-{E}_{T})\}}^{1/2},$$

is the distance within the depletion region, where traps do not emit electrons because the deep energy level of the trap (*E*_*T*_) lies below the Fermi energy (*E*_*F*_), *ε*_*r*_ is the relative permittivity of GaAs (12.9), *ε*_0_ is the vacuum permittivity, and *e* is the elementary charge. The term in curly brackets in equation (2), the so-called λ-correction term, has a value of ∼1 when *U*_*R*_ is large. However, to minimize the effects of reverse bias leakage current, relatively small values of *U*_*R*_ were used in the DLTS measurements, and therefore this term was included in our calculations of *N*_*T*_. A more general calculation of *N*_*T*_, which does not include the λ-correction term, can be found in ref.^33^.

**Figure 4:**
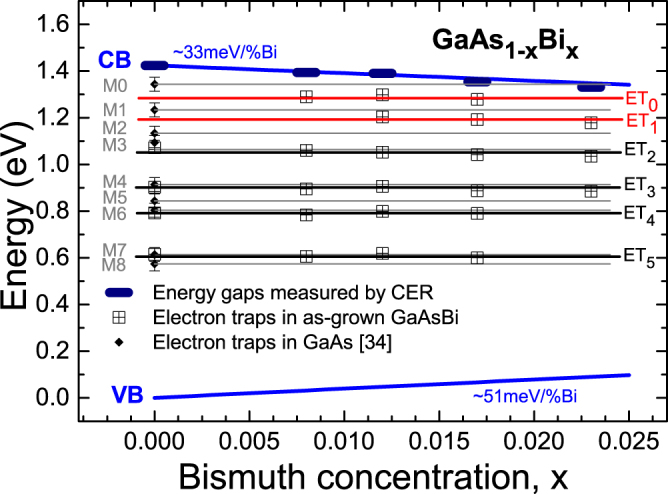
GaAsBi band diagram with the energy positions of the deep electron traps obtained from DLTS measurements. The band gap alignment was obtained from CER measurements (black thick short lines) and *ab initio* calculations^4^. The horizontal solid lines represent GaAs-like (black color) and Bi-related (red color) trap positions in the GaAsBi band gap. Moreover, the well-known native electron traps in GaAs, reported in ref.^34^, are plotted by solid diamonds, and the estimates of their positions in GaAsBi are shown by horizontal solid grey lines.

The activation energies and apparent capture cross sections of the traps, calculated from the slopes and intercepts of the Arrhenius plots shown in Fig. 2b, and their concentrations obtained from DLTS peak heights, according to equation (2), are collated in Table 2. It is immediately obvious from Table 2 that the parameters of the electron traps in the GaAs_1−*x*_Bi_*x*_ layers vary significantly with varying bismuth content. Therefore, it is rather difficult to clearly identify the same traps for all the studied samples. Specifically, the activation energies are strongly dependent on the Bi content, because of the significant reduction of the band gap energy. Recent experimental and theoretical studies^4^ showed that the incorporation of Bi atoms into a GaAs host modifies both the conduction band (CB) and the valence band (VB). It was observed that, for GaAs_1−*x*_Bi_*x*_ with 0 ≤ *x* ≤ 0.074, the CB shifts linearly at a rate of ~33 meV per % Bi, which only slightly decreases with Bi concentration. Whereas the valence band shift is clearly non-linear, being initially at a rate of ~ 51 meV/%Bi for low concentrations of Bi and then the rate is significantly reduced to ~ 20 meV/% Bi near the end of the studied composition range^4^. This specific property is in accordance with the widely accepted BAC model^8^ and similar to the band structure of dilute nitride GaAs_1−*x*_N_*x*_ alloys. However, in the latter case the strong reduction of the band gap (~100 meV/%N), with increasing N concentration, originates mostly from a downward shift of the CB, with the energy of the VB edge remaining almost constant. Therefore, the energy positions of deep electron traps, measured by the activation energies relative to the CB edge, should also decrease with increasing Bi (or N) concentration as the conduction band energy decreases^22^. An advantage of our approach is that we minimize these effects in trap identification by measuring the whole band gap reduction for the whole set of samples under similar conditions, and then plot a specific GaAs_1−*x*_Bi_*x*_ band diagram, as shown in Fig. 4.

**Table 2:** Activation energies (*E*_*a*_), apparent capture cross sections (*σ*_*n*_), and concentrations (*N*_*T*_) of the deep electron traps obtained from DLTS measurements.

Sample	Bi content, *x* (%)	Trap label	*E*_*a*_ (eV)	σ_n_ (cm^2^)	*N*_*T*_ (cm^−3^)
uSTF7J	0.0	J2	0.35	2.06 × 10^−15^	1.8 × 10^15^
		J3	0.52	5.76 × 10^−15^	1.3 × 10^15^
		J4	0.63	5.87 × 10^−15^	1.3 × 10^16^
		J5	0.81	3.09 × 10^−14^	1.6 × 10^15^
uSTF7G	0.8	G0	0.10	1.04 × 10^−17^	2.9 × 10^14^
		G2	0.33	2.35 × 10^−15^	2.3 × 10^14^
		G3	0.50	2.52 × 10^−15^	8.9 × 10^14^
		G4	0.61	4.53 × 10^−15^	1.4 × 10^15^
		G5	0.79	2.49 × 10^−14^	2.8 × 10^14^
uSTF7E	1.2	E0	0.09	6.62 × 10^−18^	1.3 × 10^15^
		E1	0.19	2.88 × 10^−17^	5.2 × 10^14^
		E2	0.34	3.36 × 10^−15^	1.6 × 10^15^
		E3	0.48	1.58 × 10^−15^	5.6 × 10^14^
		E4	0.59	1.69 × 10^−15^	5.9 × 10^14^
		E5	0.78	2.46 × 10^−14^	6.1 × 10^14^
uSTF7D	1.7	D0	0.07	5.57 × 10^−18^	1.0 × 10^14^
		D1	0.16	1.04 × 10^−17^	1.2 × 10^14^
		D2	0.31	2.75 × 10^−15^	4.6 × 10^14^
		D3	0.47	6.19 × 10^−16^	2.2 × 10^14^
		D4	0.56	1.15 × 10^−15^	1.2 × 10^14^
		D5	0.76	9.58 × 10^−15^	1.9 × 10^14^
uSTF7B	2.3	B1	0.15	2.68 × 10^−17^	1.8 × 10^13^
		B2	0.29	1.52 × 10^−15^	9.6 × 10^13^
		B3	0.45	6.03 × 10^−16^	3.2 × 10^13^

### The band diagram concept

Figure 4 shows the positions of the CB minimum and the VB maximum (blue lines), representing the band gap alignment determined from CER measurements (see Table 1) and *ab initio* calculations^4^, as a function of the bismuth concentration. The energy positions of deep electron traps, measured by activation energies relative to CB edge, are drawn in this figure by means of crossed squares. According to the statement presented above and arguments reported in ref.^22^, it is clearly evident that deep electron traps (ETs) of the same microscopic nature should be located along horizontal lines drawn in Fig. 4. In this way, all the deep ETs revealed in the GaAs_1−*x*_Bi_*x*_ by DLTS can be attributed to six ETs, labelled in Fig. 4 from ET_0_ to ET_5_. Four of the ETs (located at the black lines) were observed in the reference GaAs sample and, therefore, they are apparently not associated with Bi incorporation. The remaining two ETs (located at the red lines) were not observed in the GaAs reference sample and, therefore, they can be associated either directly or indirectly with the incorporation of Bi atoms into the GaAs host lattice. To clarify the relationship between the electron traps revealed in the studied GaAs_1−*x*_Bi_*x*_ layers and the electron traps typically seen in GaAs grown by MBE at standard conditions^34–37^, nine well-known GaAs-like traps, labelled M0 to M8 (according to the Lang *et al*. classification scheme)^34^, are plotted in Fig. 4 by solid diamonds and horizontal grey lines. It is now clear from Fig. 4 that four ETs (ET_2_, ET_3_, ET_4_, ET_5_), which were observed in both the reference GaAs layer and the GaAs_1−*x*_Bi_*x*_ layers, have very similar energy positions to the known electron traps in GaAs, labelled M3, M4, M6 and M7. This suggests that these traps do not involve Bi atoms in their microscopic structure. However, it should not be excluded that Bi can lead indirectly to their formation in GaAsBi alloys, because Bi incorporation considerably affects the crystal quality. The only two traps that are not observed in GaAs and must involve Bi atoms are ET_0_ and ET_1_. The actual microscopic nature of the two Bi-related defects is not known at present. In order to identify the origin and the nature of all the electron traps revealed in the GaAs_1−*x*_Bi_*x*_ layers, we need to carefully analyze the parameters of these traps and their evolution with increasing Bi concentration, and to discuss their possible atomic configurations with reference to previous works.

**Figure 5:**
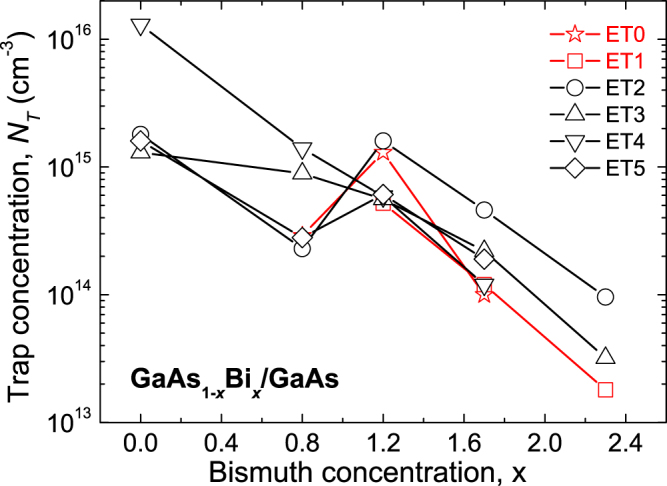
The concentration (*N*_*T*_) of the identified electron traps ET_0_ − ET_5_ versus bismuth concentration (*x*) of the n-type GaAsBi layers grown on GaAs substrates.

In Fig. 5, the ET_0_ to ET_5_ trap concentrations are plotted versus Bi concentration for the studied GaAs_1−*x*_Bi_*x*_ layers. It is immediately obvious from Fig. 5 that incorporating Bi suppresses the formation of all ETs, thus significantly reducing the total trap concentration by over two orders of magnitude. The calculated concentration of deep electron traps in the reference sample is about 1.8 × 10^16^ cm^−3^, whereas in the 2.3% Bi sample it is distinctly lower, at about 1.5 × 10^14^ cm^−3^. It is generally known that n-type GaAs layers, grown by MBE at temperatures of 550–650 °C, demonstrate low electron trap concentrations, typically in the range of 10^12^−10^13^ cm^−3^ ^34^. However, at lower growth temperatures (300–400 °C) the concentration of defects is much higher, typically in the 10^17^ to 10^18^ cm^−3^ range^16^. Higher concentrations of As_Ga_, V_Ga_ and V_As_ native defects or their complexes are expected in GaAs layers grown at lower temperatures, whereas Bi_Ga_ and Bi_As_ defects and their complexes are also predicted in GaAs_1−*x*_Bi_*x*_ layers.

### Identification of deep-level defects

**Figure 6:**
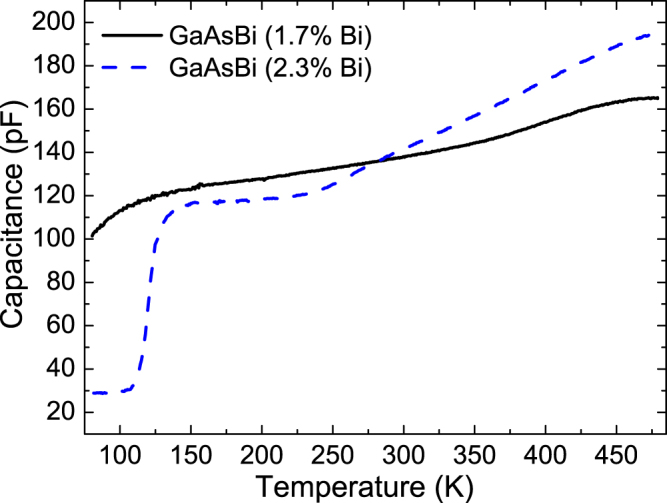
Capacitance versus temperature curves for two GaAsBi layers with 1.7% and 2.3% Bi content.

According the band diagram presented in Fig. 4 and DLTS data shown in Fig. 2a, ET_0_ and ET_1_ are not observed in the GaAs reference sample, thus they must involve Bi atoms in their microscopic structure. These traps manifest the lowest activation energies, in the ranges 0.07–0.1 eV and 0.15–0.19 eV respectively, depending on the Bi concentrations. The trap ET_0_ was observed in the samples with Bi ≤ 1.7% (i.e., traps G0, E0 and D0), whereas trap ET_1_ was observed in samples with Bi ≥ 1.2% (i.e., traps E1, D1 and B1). The absence of ET_0_ in the sample with 2.3% Bi is due to the negligible DLTS signal below ~130 K in this sample (see Fig. 2a). The weaker DLTS signal for this sample at lower temperatures is related to carrier freeze-out. Figure 6 shows the C(T) curves, i.e. temperature dependence of the capacitance, measured at 0 V bias for the samples with 1.7% and 2.3% Bi. As one can see, the freeze-out effect is stronger in the 2.3% Bi layer, where the n-type GaAs_1−*x*_Bi_*x*_ layer is fully depleted at about 130 K, i.e. the depletion width is equal to the layer thickness. Carrier freeze-out at ~200 K was previously observed in n-GaAs_1−*x*_Bi_*x*_ epitaxial layers grown at 330 °C with 1.1% Bi^19^, as well as at ~125 K in p-GaAs_1−*x*_Bi_*x*_ layers grown at 330 °C with 0.7% and 0.8% Bi and at ~100 K in p-GaAs_1−*x*_Bi_*x*_ layers grown at 370 °C with 0.8% and 0.9% Bi^15^. In this regard, we were not able to detect any DLTS signal for the sample with 2.3% Bi at low temperatures (i.e. below 130 K) as the free electrons freeze-out on donors and can no longer respond to the 1 MHz signal used for the capacitance measurements. In order to resolve the nature of the Bi-related ET_0_ and ET_1_ deep energy levels, we used the results of *ab initio* calculations reported recently by Luo *et al*.^38^, who investigated defect thermodynamics and Bi segregation in GaAsBi alloys using density functional theory (DFT). Their DFT calculations predicted defect energy levels, which are in good agreement with the measured deep energy levels of defects in n-type and p-type GaAs and GaAsBi alloys grown at low temperatures, reported recently by Mooney *et al*.^15,19,20^. According to these results, we find that the ET_0_ and ET_1_ levels revealed in the present work by DLTS, correspond reasonably well to those calculated in ref.^38^ for Bi-related pair defects, i.e. (V_Ga_ + Bi_Ga_)^−/2−^ and (As_Ga_ + Bi_Ga_)^0/1−^, respectively; the calculated energy levels of these traps are 0.1 eV and 0.18 eV below the conduction band, thus very close to our experimentally obtained values. To our knowledge, these traps have not previously been confirmed experimentally. There are only a few reports dedicated to deep level defects in n-type GaAsBi alloys and most of them claim the existence of native defects and impurities typically observed in GaAs layers grown at low temperatures. Recent DLTS measurements of n-type GaAs_1−*x*_Bi_x_ layers having 0 ≤ *x* ≤ 0.012 and GaAs grown by MBE at substrate temperatures between 300 and 400 °C showed that the dominant traps in dilute GaAsBi layers are defect complexes involving As_Ga_ as expected for MBE growth at low temperatures^19,20^. However, the authors also observed a single electron trap labeled A’ with a deep level located at *E*_*C*_ − 0.12 eV (similar to the trap ET_0_) only in the GaAsBi sample having 0.7% Bi and they claimed that this defect may involve Bi as a constituent.

On the other hand, the trap ET_2_ is observed both in the GaAs reference sample and in all the GaAs_1−*x*_Bi_*x*_ samples (i.e., traps J2, G2, E2, D2, and B2), being the dominant electron trap (with a concentration of about 9.6 × 10^13^ cm^−3^) in the 2.3% Bi sample. The deep level location of this trap suggests its attribution to the trap M3, usually observed in GaAs grown by MBE^34^. The signature of the trap M3 (*E*_*C*_ *−* 0.33 eV) is also very close to the EL6 level, according to the EL classification scheme proposed by Martin *et al*.^39^. The M3 (EL6) trap is commonly associated with a native pair defect, consisting of two point defects: the arsenic antisite (As_Ga_), and unknown X_EL6_^40^, for which the gallium vacancy (V_Ga_)^41^ and arsenic vacancy (V_As_)^42^ were proposed as the possible candidates. Moreover, the EL6 trap was also previously identified as divacancy complex defect (V_Ga_ − V_As_) by Fang *et al*.^43^, who reported a decreasing of the EL6 concentration and an increasing of the concentration of another native point defect called EL2 under thermal annealing at 800 °C for 60 min. The authors suggested that a part of EL6 was transformed into EL2. It has been also regarded by some authors as the major recombination center in GaAs^37,43^. Because ET_2_ is the dominant defect level detected in our 2.3% Bi sample, we cannot exclude its attribution to Bi-related defects. Indeed, DFT calculations performed by Luo *et al*.^38^ indicate the existence of another complex defect (V_Ga_ + Bi_As_)^2−/3−^ with an activation energy of 0.36 eV, which is very close to the ET_2_ level. The predicted defect energy level of (V_Ga_ + Bi_As_)^2−/3−^ is also reasonably consistent with recent DLTS experiments which estimated the energy position of the majority-electron trap E3 at about 0.23–0.28 eV below the conduction band in n-type GaAs_1−*x*_Bi_*x*_ layers with 0.3% Bi, grown by MBE under intense UV illumination^44^.

Another deep-level defect detected in all of the present samples is called ET_3_. The traps attributed to the level ET_3_ are trap J3 observed in the GaAs reference sample and traps G3, E3, D3, and B3 found in the GaAs_1−*x*_Bi_*x*_ samples. However, trap ET_3_ manifests threefold lower concentration in the sample with 2.3% Bi (labeled B3 in this sample), in comparison to the trap ET_2_ (labeled B2 in this sample) and equal to about 3.2 × 10^13^ cm^−3^. It can be attributed to trap M4, with a deep-level located at about 0.52 eV below CB edge in GaAs. M4 has very similar parameters to the EL4 level, commonly observed in GaAs grown by MBE or MOVPE^34–37,39^. The (M4) EL4 level is generally associated with impurity-defect complexes involving arsenic vacancies (V_As_)^35^. It is worth noting that the formation of the impurity-related trap M4 is controlled by the impurity incorporation process, which is strongly dependent on the growth temperature. At growth temperatures above 600 °C, this trap is usually not observed in GaAs, due to the very low concentration of impurities incorporated in the crystal at higher temperatures^35^.

Next, trap ET_4_ is probably attributable to trap M6 (*E*_*C*_ − 0.62 eV), which is unique to MBE-grown GaAs material, and it is also not relatable to any of the EL series of traps^39^. It is the dominant deep electron trap (see the trap J4 in Fig. 2a) observed in our GaAs reference sample, having a concentration of about 1.3 × 10^16^ cm^−3^. We note also that the incorporation of Bi strongly suppresses the formation of this trap in GaAs_1−*x*_Bi_*x*_ layers (i.e., traps G4, E4, and D4), thus reducing its concentration to about 1.2 × 10^14^ cm^−3^ in the sample with 1.7% Bi and finally being completely removed in the sample with 2.3% Bi. Few reports have been dedicated to this trap so far. Nevertheless, it was considered by some authors that trap M6 could be related to native or impurity-related defect complexes containing both gallium and arsenic vacancies (i.e., V_As_−V_Ga_ or V_As_−X−V_Ga_, where X might be interstitial or impurity related)^45^.

The deepest electron trap ET_5_ was also observed in the reference sample (i.e. trap J5) and three GaAs_1−*x*_Bi_*x*_ samples having 0 ≤ *x* ≤ 0.017 (i.e., traps G5, E5, and D5). The ET_5_ trap concentration decreases from 1.6 × 10^15^ cm^−3^ in the GaAs layer to 1.9 × 10^14^ cm^−3^ in the 1.7% Bi layer. It was not detected in the layer having 2.3% Bi. According to the band diagram, presented in Fig. 3, ET_5_ is most probably related to trap M7 (*E*_*C*_ − 0.81 eV), which manifests its parameters very close to the well-known deep-level called EL2^39^. In the last three decades, the EL2 level has been one of the most important and widely investigated native deep-level defects in bulk and epitaxial GaAs grown by different techniques^46^. It is generally accepted that EL2 is related to the isolated arsenic antisite defect (As_Ga_) or a defect complex involving As_Ga_ with an arsenic interstitial (As_i_) as a neighbour. The importance of this defect results from the fact that EL2 basically controls the electrical and optical properties of GaAs, and its existence makes it possible to achieve thermally stable semi-insulating GaAs, which is a key material in integrated circuit technology.

### Optical properties

It is well known that electron traps, besides electrical properties, can influence and control the optical properties of a given alloy. In order to study this issue, CER and PL measurements were performed on the GaAsBi samples. Due to the absorption-like character of CER spectroscopy this technique is not sensitive to point defects and therefore the band gap and the strain-induced splitting of the heavy- and light-hole bands can be determined from these measurements.

**Figure 7:**
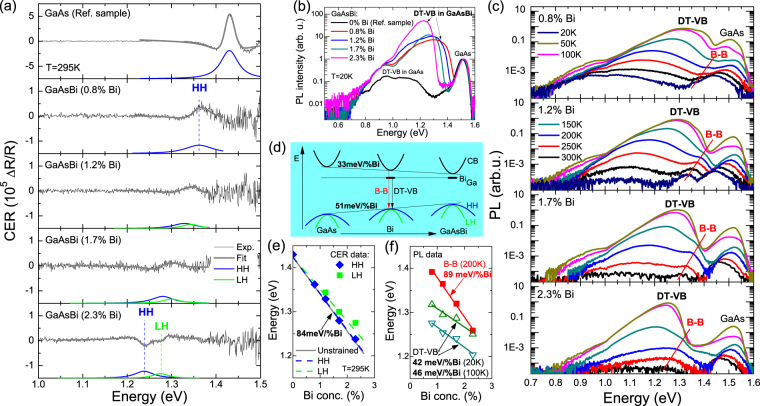
(**a**) Room temperature CER spectra of the GaAsBi layers. (**b**) Low temperature PL spectra of the GaAsBi layers. (**c**) Temperature dependent PL spectra of the GaAsBi layers. (**d**) Diagram of Bi-induced changes in the conduction and valence bands together with the energy position of DT and a schematic illustration of the optical transitions observed in the PL spectra (DT-VB and B-B transition). (**e**) Energies of the HH and LH transitions obtained from CER measurements (solid squares) and theoretical predictions (solid and dashed lines). (**f**) Energies of the DT-VB and B-B emission peaks obtained for GaAsBi samples of various Bi concentrations.

In Fig. 7a it is visible that with the increase in Bi concentration the GaAsBi-related signal redshifts and splits into two components: one related to the optical transition between the heavy-hole subband and the conduction band (HH transition), the other related to the optical transition between the light-hole subband and the conduction band (LH transition). The energies of these transitions are determined by fitting the CER spectra by Aspnes’s formula^25^ like in previous papers^4^. These energies are in very good agreement with theoretical predictions obtained according to ref.^4^, see solid squares and dashed lines in Fig. 7e. This means that the Bi-related reduction of band gap in these samples equals ~84 mV/%Bi and is significantly larger than the Bi-related shift of the PL band which is associated with donor traps. This band is clearly visible in Fig. 7b,c and is marked as DT-VB since it is the radiative recombination between donor traps (DT) and the valence band (VB). A similar emission band was observed for GaNAs alloys^22–24^. Beside DT-VB emission a band-to-band (B-B) recombination is visible in the PL spectra recorded at higher temperatures, see Fig. 7c. The Bi-related shift of the B-B transition equals 89 meV/%Bi and is very consistent with the Bi-related band gap reduction observed in the CER measurements. The spectral shift of the maximum of the DT-VB transition with increasing Bi concentration is almost two times smaller and equals 42 and 46 meV/%Bi at 20 and 100 K, respectively. These values are very close to the Bi-related shift of the valence band which equals 51 meV/%Bi. Therefore this emission is attributed to the radiative recombination between the donor traps identified in the DLTS measurements and the valence band as schematically shown in Fig. 7d. The large broadening of the DT-VB emission is fully understood in this case since the donor traps identified in the GaAsBi layers have different activation energies and their energies fit very well to the spectrum of the DT-VB emission. Moreover it is important to note that the DT-VB transitions have a non-radiative character above some temperature (T > 250 K) and therefore this transition is not observed in PL spectra at room temperature. Very similar behavior of the DT-VB emission was observed for GaAsN layers^22^. This means that the observed DTs, besides the control of electrical properties, influence the efficiency of PL.

### Summary

In summary, we investigated deep-level defects in MBE grown n-type GaAs_1−*x*_Bi_*x*_ layers with 0 < *x* < 0.023 using DLTS. We find that incorporating Bi suppresses the formation of GaAs-like electron traps, thus reducing the total trap concentration in dilute GaAsBi layers by over two orders of magnitude compared to GaAs grown under the same conditions. In order to distinguish between Bi- and host-related deep electron traps and to identify their possible origin, we used the GaAsBi band gap diagram to correlate the activation energies of the electron traps in samples with different Bi contents. On the basis of this diagram and under the support of recent theoretical calculations, at least two Bi-related traps were revealed and associated with Bi pair defects, i.e. (V_Ga_ + Bi_Ga_)^−/2−^ and (As_Ga_ + Bi_Ga_)^0/1−^. In addition, the Bi-dependence of the energy gap and defect-related emission characteristics of the GaAsBi layers have been studied by CER and PL, respectively. These measurements indicate that the revealed defects, beside the control of electrical properties, also influence the photoluminescence properties of GaAsBi alloys.

## Methods

### Sample growth

Samples were grown in an Omicron MBE-STM system on epi-ready n + -GaAs substrates (2′′ diameter wafers of 400 μm thickness, cleaved to 11.8 mm × 11.3 mm). For each sample, a 500 nm thick n + -GaAs buffer layer (Si-doped at about 1.66 × 10^18^ cm^−3^) was grown at a nominal growth temperature equal to 583 °C, using As_2_ with an As:Ga flux ratio of approximately 1.5:1. Subsequently, the growth was interrupted to lower the substrate temperature to the desired value of 378 °C for the growth of the GaAs_1−*x*_Bi_*x*_ epilayer, using As_4_ with a reduced As:Ga flux ratio to approximately 1:1. Growth of the GaAs_1−*x*_Bi_*x*_ epitaxial layers was preceded by the deposition of a bismuth pre-layer for 30 sec., prior to opening the gallium cell. The growth rate varied between 0.607 ML/s and 0.640 ML/s during the series. This was caused by the gradual depletion of the gallium cell and some daily variation due to the cell being cooled to its standby temperature (400 °C) when not in use (operating temperature: 1000 °C). Typically, the growth rate was measured after the growth of the ~500 nm buffer by growing 10–20 nm of n + -GaAs on top of the buffer, while measuring the oscillations in the intensity of the reflected high energy electron diffraction (RHEED) pattern. Coupled with the variation in growth rate, this resulted in some variation of the thickness of the n + -GaAs buffer throughout the series, with thicknesses of 500–530 nm.

### Electrical measurements

For electrical characterization, Au/Ge ohmic contacts were deposited on the whole back side of each sample, and annealing was performed at 350 °C in an Ar atmosphere for 5 min. Subsequently, circular Au Schottky contacts (0.5 mm in diameter) were deposited in vacuum through a shadow mask by electrolithography on the top side of the samples. Current-voltage (I-V) measurements were carried out to select the diodes with the lowest leakage current (<2 μA at −1 V) for the DLTS measurements. For DLTS experiments, samples were mounted in a liquid nitrogen cooled Janis VPF-475 cryostat, equipped with a Lakeshore 331 temperature controller, Boonton 7200 capacitance bridge (operating at 1 MHz) and 2601 A Keithley SMU instrument.

### Optical measurements

For CER measurements, the samples were mounted in a capacitor with a semi-transparent electrode made from a copper-wire mesh. This electrode was kept at a distance of ~0.5 mm from the sample surface while the sample itself was fixed on the bottom copper electrode. A maximum peak-to-peak alternating voltage of ~3 kV was applied. The frequency of the AC voltage was 285 Hz. Phase sensitive detection of the CER signal was made using a lock-in amplifier. Other relevant details of CER measurements are described in ref.^47^. For PL measurements, the samples were mounted in a closed-cycle refrigerator and cooled down to 20 K. The 532 nm line of a semiconductor laser was used as an excitation source (providing an excitation power density of 50 W/cm^2^) and a GaInAs photodiode as a detector. The standard lock-in amplifier technique was used to measure PL signal.
